# Atopy in children with juvenile systemic lupus erythematosus is associated with severe disease

**DOI:** 10.1371/journal.pone.0177774

**Published:** 2017-05-17

**Authors:** Ruru Guo, Yanqing Zhou, Liangjing Lu, Lanfang Cao, Junjia Cao

**Affiliations:** 1 Department of Rheumatology, Renji Hospital, School of Medicine, Shanghai Jiaotong University, Shanghai, P. R. China; 2 Department of Pediatrics, Central hospital of Jiading, Shanghai, P.R.China; 3 Department of Pediatrics, Renji Hospital, School of Medicine, Shanghai Jiaotong University, Shanghai, P. R. China; IMAGINE, FRANCE

## Abstract

The influence of co-existing atopy on the prognosis of juvenile systemic lupus erythematosus (JSLE) was assessed in this study. Patients diagnosed with JSLE between October 2005 and April 2016 were enrolled in a prospective study and followed up for 2 years. Management of patients was evaluated using the systemic lupus erythematosus disease activity index 2000 (SLEDAI-2K) score and laboratory variables. Eighty JSLE patients were enrolled at diagnosis and divided into those with (*n* = 35) and without (n = 45) atopy. When compared with the non-atopic group, atopic patients showed higher SLEDAI-2K score at disease onset (16.09 *vs*. 11.18), higher erythrocyte sedimentation rate (52.89 *vs*. 38.27 mm/h), higher percentage of total B-cells (25.85 *vs*. 19.51%), lower percentage (7.26 *vs*. 9.03%) and cytotoxicity (9.92 *vs*. 11.32%) of natural killer cells, and lower complement C3 (0.51 *vs*. 0.69 g/L) (all *p*<0.05). At 1, 3, 6, 12, 18, and 24 months, JSLE patients with atopy reached higher SLEDAI-2K score and lower ΔSLEDAI-2K improvement rate (at 1 month, 8.34 *vs*. 4.71 and 43.63 *vs*. 57.95%, respectively; at 3 months, 8.57 *vs*. 2.62 and 48.39 *vs*. 75.10%, respectively; at 6 months, 6.91 *vs*. 2.38 and 53.59 *vs*. 77.26%, respectively; at 12 months, 4.71 *vs*. 1.80 and 69.54 *vs*. 84.10%, respectively; at 18 months, 4.66 *vs*. 2.02 and 68.14 *vs*. 82.93%, respectively; at 24 months, 8.57 *vs*. 2.62 and 70.00 *vs*. 81.88%, respectively; all *p*<0.05). During the 24 months of follow-up, the total number of disease flares was higher in JSLE patients with co-existing atopy (3.77 *vs*. 1.51, *p*<0.05), and the atopic group needed much more time to reach the stable condition of the disease (6.88 *vs*. 4.65 months, *p*<0.05). JSLE patients combined with co-existing atopy had more severe disease at diagnosis and poorer outcomes than JSLE patients without atopy.

## Introduction

Juvenile systemic lupus erythematosus (JSLE) is a multisystem autoimmune disorder that results from complex abnormalities of the innate and adaptive immune systems, resulting in inflammation and potential damage to a variety of organs. For reasons that are currently not well elucidated, the disease course and phenotype associated with SLE, although quite variable, are generally more severe when diagnosed during childhood [1, 2]. Prevalence rates of JSLE have varied from 4 to 250 per 100,000 population [3–5]; JSLE accounts for 15–20% of all cases of SLE, with the onset most frequently diagnosed between the ages of 12 and 14 years [1]. This wide prevalence rate is probably, at least partially, attributable to the availability of better diagnostic tests, increasing awareness of the disease, the establishment of standardized diagnostic criteria, data from various ethnic groups, and geographic differences. Many factors, like genetic predisposition, environmental factors, and abnormalities in the complicated regulatory network of the immune system, are speculated to have an important role in the pathogenesis of JSLE [1, 6].

Atopy refers to an inherited tendency to produce immunoglobulin E (IgE) antibodies in response to small amounts of common environmental proteins, such as pollens, house dust mites, and food allergens. The presence of atopy in an individual is associated with an increased risk of developing one or more of atopic diseases including atopic dermatitis, asthma, and allergic rhinoconjunctivitis/hay fever (and food allergy). However, atopy can be present in the form of asymptomatic sensitization to one or more allergens, which means that an individual with confirmed allergic sensitization does not exhibit clinical allergy [7]. The prevalence of atopy in childhood has increased since the latter part of the 20th century [7, 8]. And there are very different atopy rates that have been reported in different regions [9, 10]. Studies have reported atopy rates that range from 20.5% in Germany [11] to 57.7% in Hong Kong [12]. The wide range of prevalence of atopy may due to the international variability and regional differences [9,10]. Atopy is associated with an aberrant response to allergens through IgE production by antigen-specific Th2 cells and B cells [13].

JSLE and atopy are chronic immune-mediated diseases characterized by inflammatory responses which, although considered to be distinct clinical immunological conditions, share important epidemiological similarities [14]. These similarities seem to imply that autoimmune diseases and allergies share a common etiology. Some indications for the plausibility of underlying similarities are the rise in prevalence of both disease groups in the 20th century [15]. Previous researches showed epidemiological correlations and a substantial pathophysiological relationship between atopy and SLE [2, 16, 17]. Furthermore, many recent discoveries have challenged that there is no connection between atopic diseases and autoimmune disorders and even suggested that autoimmune and atopic diseases may be risk factors for each other, contributing to the high prevalence of both diseases [2, 13, 16, 18,19].

Recently, many clinical studies have challenged the concept that atopy and autoimmunity are mutually exclusive states [20]. However, there is no data about the effect of co-existing atopy on SLE disease activity and prognosis in both children and adults. In this study, we are the first to assess the influence of co-existing atopy in children with JSLE on outcome.

## Patients and methods

The diagnosis of JSLE and atopy was performed by rheumatologists and allergists, respectively, at Renji Hospital in Shanghai between October 2005 and April 2016. Eighty JSLE patients had a mean age of 11.5 years (range 5–16 years). SLE diagnosis was made according to the American College of Rheumatology criteria for the classification of systemic lupus erythematosus, published in 1997 [21], with disease onset before 16 years of age. There are exclusion criteria for this study, including infectious diseases, leukemia, lymphoma, and other connective tissue diseases. The study was conducted according to the Declaration of Helsinki, and ethics committee approval for this study was obtained at Renji Hospital, School of Medicine, Shanghai Jiaotong University. Guardians gave written informed consent on behalf of the children according to the guidelines of the ethical research committee of the hospital prior to their inclusion in the present study.

All patients were assessed monthly by pediatric rheumatologists and allergists during the trial. As described in detail previously [19], a standardized data collection protocol (S1 Dataset) was used for all patients, including basic information, medical history, history of autoimmune diseases in a first-degree relative, and any individual or family history of atopy. The clinical assessments of each patient described in detail in our previous study [19] were included and SLE disease activity index 2000 (SLEDAI-2K) [22] is used to reflect the change of disease activity. SLEDAI-2K may range from 0 to 105 (0 = no activity; 1–5 = mild activity; 6–10 = moderate activity; 11–19 = high activity; ≥20 = very high activity; flare = increase in score by ≥3) [22]. To assess and evaluate the changes of SLEDAI-2K score during the 24-month follow-up, we introduced the term “improvement rate” of SLEDAI-2K score, which is expressed by ΔSLEDAI-2K [ΔSLEDAI-2K = (X0-X)/X0; X0 = SLEDAI-2K at the baseline; X = SLEDAI-2K at the 1st, 3rd, 6th, 12th, 18th, and 24th months of the follow-up, respectively]. A diagnosis of atopy at study entry was made in the context of a positive antigen-specific IgE concentration of >0.70 kU/L or positive skin prick test (SPT) results [23]. All JSLE patients with atopy were also assessed by pediatric allergists. The clinical assessments of atopy focused on the symptoms of allergy related diseases and changes of total IgE at different points of follow-up. During the trial, eight patients with manifestations of clinical allergy and antianaphylaxis treatment were excluded.

### Laboratory tests

Antigen-specific IgE antibodies were measured with Allergen ImmunoCAP using the Pharmacia UniCAP System and the method is also described in detail in our previous work [19]. Serum total IgE (normal range, 0–100 IU/ml), erythrocyte sedimentation rate (ESR) (normal range, 0–20 mm/h), natural killer (NK) cell cytotoxicity (which was measured with lactate dehydrogenase release assay [24]) (normal range 15–25%), lymphocyte count, complete blood cell count, serum urea and creatinine, urinalysis, and 24-hour urine protein excretion were measured at Renji Hospital. Complement levels [CH50 (normal range, 20-50u/ml), C3 (normal range, 0.9–1.8g/l), and C4 (normal range, 0.1–0.4g/l)], antinuclear antibodies (ANA) (normal range, <1:160), extractable nuclear antigen (ENA) (normal range, <1:160), and anti-double-stranded DNA autoantibodies (normal range, 0–7.0 IU/ml) were determined at each of the centers involved, and the cut-off values were considered valid.

All patients underwent a SPT using a standardized protocol, which was described in detail in our previous study [19]. A positively scored wheal diameter was ≥3 mm larger than the negative control.

Lactate dehydrogenase release-cytotoxicity assay kit (Abcam, Germany) [24]: human peripheral blood mononuclear cells (PBMC) were isolated, and resuspended in RPMI 1640 supplemented with 10% fetal calf serum (FCS) at a density of 2×10^4^ cells/well in a 96-well plate. Prepare the following samples individually in a 96-well plate: background control (200ul culture medium per well with no cells), low control (200ul cells), high control (200ul cells containing 1% Triton X-100) and test sample (200ul cells adding test substances). Add 2×10^4^ cells/well in 200ul assay medium into triplicate wells and incubate cells in an incubator (5% CO_2_, 90% humidity, 37°C) for 2h. Transfer 100u/well supernatant into corresponding wells of an optically clear 96-plate and add 100ul reaction mixture to each well and incubate for up to 30 min at room temperature. Finally, measure the absorbance of all samples at 490nm using a microtiter plate reader. The results will be expressed by cytotoxicity (%) = (test sample-low control)/(high control-low control) ×100.

### Statistical analysis

Categorical data were expressed by counts or percentages and compared between different groups using the chi-square test or Fisher’s exact test. The continuous variables were presented as mean ± standard deviation and analyzed by Student’s t test or Mann–Whitney U-test as appropriate. Correlation analyses were performed using the Spearman’s rank correlation. A p-value of <0.05 was considered as statistically significant. All analyses were performed using SPSS software version 13.0 (SPSS Inc.; Chicago, IL, USA).

## Results

This prospective study enrolled 80 JSLE patients diagnosed by rheumatologists at the Renji Hospital between October 2005 and April 2016. The 14 boys and 66 girls had a mean current age of 13.19±2.59 years (range 4–16 years) and a mean age at diagnosis of 11.5 years (range 5–16 years). Girl patients outnumbered boys with a ratio of 4.7:1. The JSLE patients were divided into those with (*n* = 35) and without (*n* = 45) atopy. The majority of atopic patients were positive for inhaled allergens (96.15%), food allergens (69.23%), and SPT (82.13%). Table 1 shows the characteristics of atopy. Clinical allergy and antianaphylaxis treatment may influence the results of this research; thus, all the 8 patients who develop atopy-related disease were excluded.

**Table 1:** Clinical characteristics of atopy in children with JSLE.

Characteristic	Subjects (*n* = 35)
**Positive serum specific IgE of inhaled allergens, n**	27
Mold, n	3
Pollen, n	0
Dust mites, n	12
Herbs, n	5
Cockroaches, n	2
Latex, n	0
Cat hair, n	4
Dog hair, n	1
**Positive serum specific IgE of food allergens, n**	21
Milk, n	8
Eggs, n	9
Shrimp, n	3
Crabs, n	1

At disease onset, the atopic group exhibited higher mean SLEDAI-2K score, higher anti-double-stranded DNA, higher ESR, higher total serum IgE, and lower complement C3 and C4 when compared with the non-atopic group (all *p*<0.05; Tables 2 and 3). Although the atopic group showed higher total serum IgE, the number of patients with total IgE>100 IU/ml in both groups was not significantly different. In particular, B lymphocyte as the important part of the humoral immunity was measured and the percentage of B cell population in the atopic group was higher than that in the non-atopic group (25.85 vs. 19.51%, *p* = 0.038), whereas the NK cell cytotoxicity was evidently lower in the atopic group (*p*<0.05). Glucocorticoids and immunosuppressive agents are the mainstay of treatment. At the study enrollment, the cumulative glucocorticoid (prednisolone-equivalent steroid dose) calculated by unit body weight and the number of combined immunosuppressors received by atopic patients were higher compared to those received by non-atopic patients, with obvious statistical significance (Table 3).

**Table 2:** Demographics and characteristic of JSLE at the baseline.

Characteristic	Non-atopy(*n* = 45)	Atopy(*n* = 35)	*p*-value
**Age at diagnosis** ^a^, **year**	11.91 (2.44)	10.89 (2.87)	0.088
**Gender girls, *n* (%)**^b^	40 (88.9)	26 (74.3)	0.088
**Proportion of familial history of autoimmune diseases**	4 (8.9)	2 (5.7)	0.915
**Time from onset to diagnosis** ^a^, **month**	7.16 (12.43)	4.27 (4.13)	0.149
**Fever, *n* (%)**^b^	23 (51.1)	22 (62.9)	0.293
**No. of involvement organ** ^a^	2.56 (1.22)	3.00 (1.39)	0.132
**ANA, positive *n* (%)**^b^	40 (88.9)	34 (97.1)	0.336
**ENA, positive *n* (%)**^b^	29 (64.4)	23 (65.7)	0.906
**SLEDAI-2K score** ^a^	11.18 (4.70)	16.09 (5.65)	0.000*
**Classification of SLEDAI-2K**^c^			0.000*
No activity, *n* (%)	0 (0.0)	0 (0.0)	
Mild activity, *n* (%)	7 (15.6)	0 (0.0)	
Moderate activity, *n* (%)	16 (35.6)	4 (11.4)	
High activity, *n* (%)	20 (44.4)	23 (65.7)	
Very high activity, *n* (%)	2 (4.4)	8 (22.9)	
**CRP (mg/l)**^a^	4.33 (3.58)	4.09 (3.11)	0.753
**ESR (mm/h)** ^a^	38.27 (30.58)	52.89 (32.39)	0.042*
**CH50 (U/ml)** ^a^	30.12 (18.92)	20.96 (19.36)	0.037*
**Complement C3 (g/L)** ^a^	0.69 (0.34)	0.51 (0.27)	0.011*
**Complement C4 (g/L)** ^a^	0.12 (0.15)	0.06 (0.05)	0.029*

All values are mean (SD), unless otherwise specified.

^a^ Mean (standard deviation), between group comparison (Student’s t test)

^b^ Frequency/total, n (%), between group comparisons (chi-square test)

^c^ Frequency/total, n (%), between group comparisons (Fisher’s exact test)

*p<0.05 indicates a significant difference between the different groups

**Table 3:** The characteristic of immune system and medications at the baseline.

Characteristic	Non-atopy(*n* = 45)	Atopy(*n* = 35)	*p*-value
**Total serum IgE (IU/ml)** ^a^	119.66 (68.37)	266.99 (232.00)	0.001*
**Total serum IgE>100IU/ml, *n* (%)**^b^	23 (51.1)	24 (68.6)	0.169
**CD3**^**+**^**T cell percentage (%)**^a^	65.90 (13.39)	64.01 (11.07)	0.502
**CD3**^**+**^**CD4**^**+**^**T cell percentage (%)**^a^	27.51 (8.54)	25.61 (7.07)	0.292
**CD3**^**+**^**CD8**^**+**^**T cell percentage (%)**^a^	34.47 (11.29)	35.19 (7.96)	0.741
**T helper/T suppressor** ^a^	0.94 (0.42)	0.77 (0.31)	0.060
**Total B cell percentage (%)**^a^	19.51 (14.82)	25.85 (11.20)	0.038*
**NK cell percentage (%)**^a^	9.03 (3.85)	7.26 (3.37)	0.035*
**NK cell cytotoxicity (%)**^a^	11.32 (2.23)	9.92 (1.33)	0.001*
**NEUTR (%)**^a^	65.67 (11.49)	47.27 (14.04)	0.000*
**IgA (g/L)**^a^	1.95 (0.90)	2.05 (0.88)	0.597
**IgM (g/L)**^a^	1.55 (0.81)	1.24 (0.48)	0.053
**IgG (g/L)**^a^	15.57 (8.61)	16.13 (4.81)	0.713
**GC cumulative dose (mg/kg/d)** ^a^	1.12 (0.39)	1.34 (0.32)	0.011*
**Kinds of immunosuppressor used** ^a^	0.87 (0.59)	1.43 (0.50)	0.000*

NEUTR = neutrophil granulocyte ratio. All values are mean (SD), unless otherwise specified.

^a^ Mean (standard deviation), between group comparison (Student’s t test)

^b^ Frequency/total, n (%), between group comparisons (chi-square test)

*p<0.05 indicates a significant difference between the different groups

As shown in Table 4, we used SLEDAI-2K score to evaluate the individual JSLE activity during the disease course. At the first month of follow-up, the SLEDAI-2K score of all the patients decreased but with individual variance. When compared with non-atopic patients, more atopic patients showed moderate activity. In addition, fewer patients had mild activity during the 2-year follow-up (all *p*<0.05; Tables 4 and 5). To reach a decrease of SLEDAI-2K score by 50% (SLEDAI-2K-50%) at the first month of follow-up, the cumulative glucocorticoid calculated by unit body weight taken by patients in the atopic group was higher than that in the non-atopic group, which showed statistical significance (1.02±0.27 mg/kg/d *vs*. 0.84±0.28 mg/kg/d, *p* = 0.004). Patients with atopy exhibited a lower frequency of SLEDAI-2K-50% (40.0 vs. 64.4%, *p* = 0.030). At the 1st, 3rd, 6th, 12th, 18th, and 24th months, JSLE patients with atopy had higher SLEDAI-2K score, cumulative glucocorticoid calculated by unit body weight, and kinds of immunosuppressors used (all *p*<0.05; Tables 4 and 5). Comparison between the two groups indicated a significantly lower complement C3 and NK cell cytotoxicity in the atopic group during the follow-up (all *p*<0.05; Tables 4–6).

**Table 4:** Laboratory features and clinical assessment of JSLE patients at the first, 3rd, 6th, and 12th month of follow-up.

Time point	Non-atopy(*n* = 45)	Atopy(*n* = 35)	*p*-value
**1st month follow-up**			
SLEDAI-2K score ^a^	4.71(3.47)	8.34(2.83)	0.000*
Classification of SLEDAI-2K ^b^			0.000*
No activity, *n* (%)	7 (15.6)	0 (0.0)	
Mild activity, *n* (%)	19 (42.2)	4 (11.4)	
Moderate activity, *n* (%)	16 (35.6)	21 (60.0)	
High activity, *n* (%)	3 (6.7)	10 (28.6)	
Very high activity, *n* (%)	0 (0.0)	0 (0.0)	
Anti-ds-DNA (IU/ml) ^a^	27.86(14.19)	59.98(38.77)	0.000*
ESR (mm/h) ^a^	16.29(14.18)	24.43(19.61)	0.034*
CH50 (U/ml) ^a^	39.99(14.80)	41.07(12.90)	0.733
Complement C3 (g/L) ^a^	0.95(0.28)	0.78(0.17)	0.002*
Complement C4 (g/L) ^a^	0.14(0.07)	0.10(0.06)	0.008*
NK cell cytotoxicity (%)^a^	11.17(1.97)	10.09(1.18)	0.003*
SLEDAI-2K (-50%), *n* (%)^c^	29(64.4)	14(40.0)	0.030*
GC cumulative dose (mg/kg/d) ^a^	0.84(0.28)	1.02(0.27)	0.004*
Kinds of immunosuppressor used ^a^	1.07(0.69)	1.37(0.49)	0.029*
**3rd month follow-up**			
SLEDAI-2K score ^a^	2.62(2.18)	8.57(5.62)	0.000*
Classification of SLEDAI-2K ^b^			0.000*
No activity, *n* (%)	10 (22.2)	0 (0.0)	
Mild activity, *n* (%)	29 (64.4)	12 (34.3)	
Moderate activity, *n* (%)	6 (13.3)	14 (40.0)	
High activity, *n* (%)	0 (0.0)	7 (20.0)	
Very high activity, *n* (%)	0 (0.0)	2 (5.7)	
Anti-ds-DNA (IU/ml) ^a^	11.94(7.80)	13.60(11.12)	0.434
ESR (mm/h) ^a^	21.11(18.70)	34.51(30.04)	0.017*
CH50 (U/ml) ^a^	45.75(14.28)	46.72(13.09)	0.754
Complement C3 (g/L) ^a^	1.06(0.30)	0.91(0.19)	0.005*
Complement C4 (g/L) ^a^	0.19(0.07)	0.17(0.14)	0.424
NK cell cytotoxicity (%)^a^	11.16(1.80)	10.20(1.31)	0.010*
GC cumulative dose (mg/kg/d) ^a^	0.59(0.23)	0.71(0.22)	0.013*
Kinds of immunosuppressor used ^a^	1.22(0.60)	1.49(0.51)	0.040*
**6th month follow-up**			
SLEDAI-2K score ^a^	2.38(2.42)	6.91(4.26)	0.000*
Classification of SLEDAI-2K ^b^			0.000*
No activity, *n* (%)	17 (37.8)	3 (8.6)	
Mild activity, *n* (%)	20 (44.4)	10 (28.6)	
Moderate activity, *n* (%)	8 (17.8)	16 (45.7)	
High activity, *n* (%)	0 (0.0)	6 (17.1)	
Very high activity, *n* (%)	0 (0.0)	0 (0.0)	
Anti-ds-DNA (IU/ml) ^a^	12.95(11.46)	13.32(13.11)	0.893
ESR (mm/h) ^a^	13.41(10.98)	15.29(12.69)	0.475
CH50 (U/ml) ^a^	45.44(14.10)	46.07(14.90)	0.847
Complement C3 (g/L) ^a^	1.04(0.23)	0.83(0.35)	0.002*
Complement C4 (g/L) ^a^	0.18(0.07)	0.14(0.08)	0.022*
NK cell activity (%)^a^	11.11(1.84)	10.38(1.14)	0.032*
GC cumulative dose (mg/kg/d) ^a^	0.36(0.18)	0.52(0.28)	0.005*
Kinds of immunosuppressor used ^a^	1.33(0.60)	1.63(0.55)	0.027*
**12th month follow-up**			
SLEDAI-2K score ^a^	1.80(1.97)	4.71(3.61)	0.000*
Classification of SLEDAI-2K ^b^			0.000*
No activity, *n* (%)	20 (44.4)	6 (17.1)	
Mild activity, *n* (%)	23 (51.1)	14 (40.0)	
Moderate activity, *n* (%)	2 (4.4)	13 (37.1)	
High activity, *n* (%)	0 (0.0)	2 (5.7)	
Very high activity, *n* (%)	0 (0.0)	0 (0.0)	
Anti-ds-DNA (IU/ml) ^a^	11.39(8.70)	17.06(15.37)	0.040*
ESR (mm/h) ^a^	13.84(10.07)	18.09(16.13)	0.154
CH50 (U/ml) ^a^	45.95(11.39)	46.87(10.77)	0.715
Complement C3 (g/L) ^a^	1.08(0.15)	0.99(0.20)	0.028*
Complement C4 (g/L) ^a^	0.19(0.10)	0.17(0.07)	0.385
NK cell cytotoxicity (%)^a^	11.45(1.33)	10.47(1.58)	0.003*
GC cumulative dose (mg/kg/d) ^a^	0.26(0.13)	0.38(0.26)	0.010*
Kinds of immunosuppressor used ^a^	1.22(0.60)	1.74(0.56)	0.000*

All values are mean (SD), unless otherwise specified.

^a^ Mean (standard deviation), between group comparison (Student’s t test)

^b^ Frequency/total, n (%), between group comparisons (Fisher’s exact test)

^c^ Frequency/total, n (%), between group comparisons (Chi-square test)

*p<0.05 indicates a significant difference between the different groups

**Table 5:** Laboratory features and clinical assessment of JSLE patients at the 18th and 24th month of follow-up.

Time point	Non-atopy(*n* = 45)	Atopy(*n* = 35)	*p*-value
**18th month follow-up**			
SLEDAI-2K score ^a^	2.02(1.98)	4.66(2.92)	0.000*
Classification of SLEDAI-2K ^b^			0.000*
No activity, *n* (%)	18 (40.0)	2 (5.7)	
Mild activity, *n* (%)	26 (57.8)	20 (57.1)	
Moderate activity, *n* (%)	1 (2.2)	12 (34.3)	
High activity, *n* (%)	0 (0.0)	1 (2.9)	
Very high activity, *n* (%)	0 (0.0)	0 (0.0)	
Anti-ds-DNA (IU/ml)^a^	13.05(13.07)	16.49(15.6)	0.287
ESR (mm/h) ^a^	13.02(10.39)	19.77(9.70)	0.004*
CH50 (U/ml) ^a^	44.93(11.30)	45.42(10.27)	0.843
Complement C3 (g/L) ^a^	1.07(0.23)	0.93(0.21)	0.010*
Complement C4 (g/L) ^a^	0.19(0.10)	0.14(0.09)	0.019*
GC cumulative dose (mg/kg/d) ^a^	0.22(0.12)	0.47(0.29)	0.000*
Kinds of immunosuppressor used ^a^	1.18(0.49)	1.66(0.59)	0.000*
**24th month follow-up**			
SLEDAI-2K score ^a^	2.62(2.18)	8.57(5.62)	0.000*
Classification of SLEDAI-2K ^b^			0.000*
No activity, *n* (%)	10 (22.2)	0 (0.0)	
Mild activity, *n* (%)	29 (64.4)	12 (34.3)	
Moderate activity, *n* (%)	6 (13.3)	14 (40.0)	
High activity, *n* (%)	0 (0.0)	7 (20.0)	
Very high activity, *n* (%)	0 (0.0)	2 (5.7)	
Anti-ds-DNA (IU/ml) ^a^	12.88(13.07)	13.56(8.42)	0.792
ESR (mm/h) ^a^	10.22(6.85)	14.34(8.04)	0.017*
CH50 (U/ml) ^a^	47.22(10.65)	45.80(10.33)	0.550
Complement C3 (g/L) ^a^	1.04(0.16)	0.85(0.21)	0.000*
Complement C4 (g/L) ^a^	0.19(0.14)	0.14(0.07)	0.103
GC cumulative dose (mg/kg/d) ^a^	0.22(0.11)	0.41(0.33)	0.001*
Kinds of immunosuppressor used ^a^	1.33(0.56)	1.69(0.58)	0.008*
No. of flares during the 2 years ^a^	1.51(1.25)	3.77(2.07)	0.000*
Time of stable condition of JSLE needed, month ^a^	4.65(3.73)	6.88(4.24)	0.034*

All values are mean (SD), unless otherwise specified.

^a^ Mean (standard deviation), between group comparison (Student’s t test)

^b^ Frequency/total, n (%), between group comparisons (Fisher’s exact test)

*p<0.05 indicates a significant difference between the different groups

**Table 6:** The characteristic of immune system at the 18th and 24th month of follow-up.

Characteristic	Non-atopy(*n* = 45)	Atopy(*n* = 35)	*p*-value
**18th month follow-up**			
Total serum IgE (IU/ml) ^a^	92.32 (73.48)	180.07 (145.59)	0.001*
Total serum IgE>100IU/ml, *n* (%)^b^	15 (33.3)	19 (54.3)	0.060
CD3^+^T cell percentage (%)^a^	70.37 (11.67)	70.50 (12.55)	0.961
CD3^+^CD4^+^T cell percentage (%)^a^	29.74 (8.51)	30.73 (8.00)	0.600
CD3^+^CD8^+^T cell percentage (%)^a^	37.20 (10.43)	37.42 (9.24)	0.922
T helper/T suppressor^a^	0.92 (0.41)	0.82 (0.32)	0.253
Total B cell percentage (%)^a^	15.19 (10.35)	20.81 (10.17)	0.017*
NK cell percentage (%)^a^	9.67 (4.00)	7.35 (3.49)	0.008*
NK cell cytotoxicity (%)^a^	11.27(1.71)	10.22(1.41)	0.004*
NEUTR (%)^a^	58.61 (11.51)	58.09 (12.15)	0.843
IgA (g/L)^a^	1.59 (0.74)	1.79 (0.88)	0.278
IgM (g/L)^a^	1.14 (0.48)	1.06 (0.71)	0.553
IgG (g/L)^a^	11.50 (7.14)	11.27 (4.44)	0.874
**24th month follow-up**			
Total serum IgE (IU/ml) ^a^	76.26 (74.60)	99.82 (78.83)	0.175
Total serum IgE>100IU/ml, *n* (%)^b^	13 (28.9)	15 (42.9)	0.240
CD3^+^T cell percentage (%)^a^	70.93 (11.65)	72.52 (13.17)	0.568
CD3^+^CD4^+^T cell percentage (%)^a^	31.55 (9.85)	31.17 (9.54)	0.862
CD3^+^CD8^+^T cell percentage (%)^a^	35.50 (9.57)	37.25 (9.37)	0.416
T helper/T suppressor ^a^	1.00 (0.41)	0.90 (0.38)	0.283
Total B cell percentage (%)^a^	14.27 (6.91)	19.22 (10.59)	0.014*
NK cell percentage (%)^a^	10.72 (4.24)	8.70 (3.17)	0.021*
NK cell cytotoxicity (%)^a^	12.10 (2.48)	10.65 (2.11)	0.007*
NEUTR (%)^a^	62.25 (12.45)	55.39 (11.85)	0.015*
IgA (g/L)^a^	1.57 (0.61)	1.67 (0.78)	0.554
IgM (g/L)^a^	1.12 (0.44)	0.98 (0.59)	0.208
IgG (g/L)^a^	10.09 (3.09)	10.31 (3.27)	0.765

NEUTR = neutrophil granulocyte ratio. All values are mean (SD), unless otherwise specified.

^a^ Mean (standard deviation), between group comparison (Student’s t test)

^b^ Frequency/total, n (%), between group comparisons (chi-square test)

*p<0.05 indicates a significant difference between the different groups

**Fig 1:**
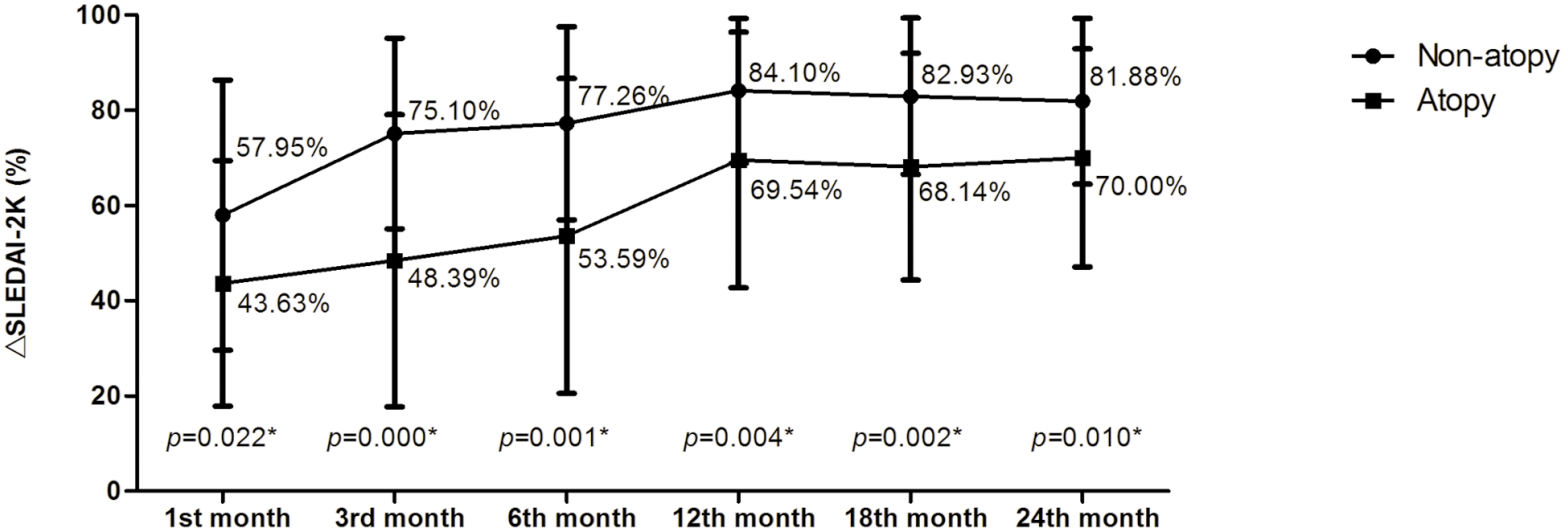
Improvement rate of SLEDAI-2K score at the first, 3rd, 6th, 12th, 18th, and 24th month of the follow-up. 1st month: ΔSLEDAI-2K1 = (X0-X1)/X0; X0 = SLEDAI-2K score at the baseline; X1 = SLEDAI-2K score at the first month of follow-up. 3rd month: ΔSLEDAI-2K3 = (X0-X3)/X0; X0 = SLEDAI-2K score at the baseline; X3 = SLEDAI-2K score at the 3rd month of follow-up. 6th month: ΔSLEDAI-2K6 = (X0-X6)/X0; X0 = SLEDAI-2K score at the baseline; X6 = SLEDAI-2K score at the 6th month of follow-up. 12th month: ΔSLEDAI-2K12 = (X0-X12)/X0; X0 = SLEDAI-2K score at the baseline; X12 = SLEDAI-2K score at the 12th month of follow-up. 18th month: ΔSLEDAI-2K18 = (X0-X18)/X0; X0 = SLEDAI-2K score at the baseline; X18 = SLEDAI-2K score at the 18th month of follow-up. 24th month: ΔSLEDAI-2K24 = (X0-X24)/X0; X0 = SLEDAI-2K score at the baseline; X24 = SLEDAI-2K score at the 24th month of follow-up. All values are mean (standard deviation), unless otherwise specified. Mean (standard deviation), between group comparisons (Student’s t test). *p<0.05 indicates a significant difference between the different groups.

To determine whether the presence of atopy in patients with JSLE was associated with an altered JSLE progression profile, we introduced the term “improvement rate” of SLEDAI-2K score, which is expressed by ΔSLEDAI-2K. The ΔSLEDAI-2K was higher in the non-atopic group at the 1st, 3rd, 6th, 12th, 18th, and 24th months (56.95 vs. 43.63%; 75.10 vs. 48.39%; 77.26 vs. 53.59%; 84.10 vs. 69.54%; 82.93 vs. 68.14%; 81.88 vs. 70.00%, respectively; all *p*<0.005; Fig 1). At the end of follow-up, the number of JSLE flares and the time to reach the stable condition of the disease were significantly higher in the atopic group than those in the non-atopic group (all *p*<0.05; Table 5). At the 18th and 24th months of follow-up, we evaluated the immune cells again. A higher number of B cell, a lower NK cell percentage, and a lower cytotoxicity of NK cell (all *p*< 0.05; Table 6) were still found. However, the total serum IgE between the two groups was not statistically different at the end of the follow-up (Table 6).

**Fig 2:**
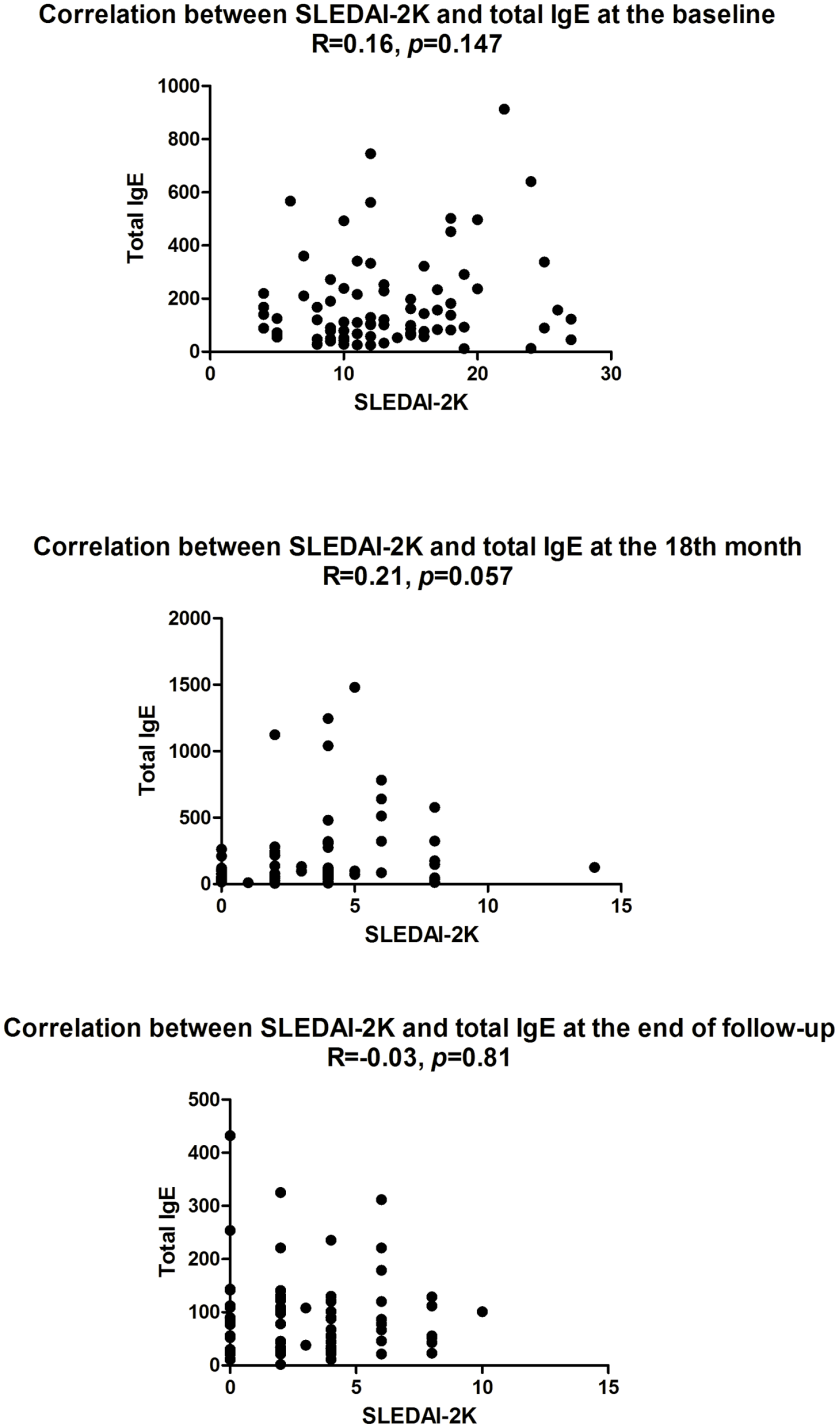
Correlations between total serum IgE and SLEDAI-2K at the baseline, 18th month and end of the follow-up.

The correlations between total serum IgE and SLEDAI-2K at the baseline, 18th month, and end of the follow-up were analyzed to evaluate possible associations between increase total serum IgE levels and JSLE disease activity. The total serum IgE levels did not statistically differ with respect to the SLEDAI-2K score (at the baseline, r = 0.16, *p* = 0.147; at the 18th month, r = 0.21, *p* = 0.057; at the end of follow-up, r = -0.03, *p* = 0.81, respectively; Fig 2)

## Discussion

This prospective cohort study of 80 patients with JSLE during a 2-year follow-up adds support to previous studies that suggest atopy and JSLE are not independent. Like adult SLE, there is a female predominance of the disease. But there is no significant difference in sex ratio between the two groups. In addition, the younger a disease appears, the more “Mendelian” its cause may be. So it is very important to address and compare the age at onset of the two groups, which can give clue to a potential monogenic autoimmune disease cause. However, there is no significant difference of the age at onset between the two groups in our study. And there is also no statistic difference in the proportion of having a familial history of autoimmune diseases between the two groups. We observed that JSLE patients with co-existing atopy at the study enrollment had higher SLEDAI-2K score, anti-double-stranded DNA, ESR, and percentage of total B cells, and lower percentage and cytotoxicity of NK cells and lower complement C3 and C4 than JSLE patients without atopy. In contrast to adult SLE, more JSLE patients had high disease activity. The results showed above support the assertion that the presence of co-existent atopy may be associated with severe JSLE and with significantly greater difficulty in the successful management of JSLE. These results are consistent with previous studies that JSLE is a more aggressive disease [2, 25], and JSLE combined with atopic disorders makes the disease more complicated. Moreover, a recent study has also shown that chronic idiopathic urticarial may be linked to JSLE and associated with lupus moderate/high disease activity [2]. One recent case report has described 4 patients with comorbid Sjogren’s syndrome and atopic dermatitis. The authors found that the abnormal sudomotor axon reflex (due to small fiber neuropathy associated with SS) accelerates the dryness of skin in atopic dermatitis, significantly contributing to pruritis and disease progression [26]. In addition, recent studies have emphasized that allergic disorders and autoimmune disease may co-exist and in fact exacerbate atopic disease, and vice versa [20, 19]. This study is the first to perform a systematic evaluation of disease parameters between JSLE with and without atopy. The prevalence of atopy in JSLE patients was 43.8%. In Shanghai, across-sectional questionnaire study found that the prevalence of asthma among children had increased from 1.90% in 1990 to 10.3% in 2011 [27,28], which was still lower compared to JSLE patients. A study of more than 12,000 patients with chronic idiopathic urticarial found that they were 17.3 times as likely to have rheumatoid arthritis, 16.3 times as likely to have SLE, and 17 times as likely to have type 1 diabetes compared to healthy controls [29]. Other studies also reported the different prevalence of atopic disorder in autoimmune diseases: 24.6% in patients with ankylosing spondylitis, 13.1% in patients with rheumatoid arthritis, and 20.5% in general pediatric population in Germany [11, 30]. The differences in atopy prevalence may be attributed to variations in the genetic constitution and environmental factors. The subjects of those previous studies are adults, while our study focuses on children. More importantly, previous studies did not exclude patients with clinical allergy manifestations. In the present study, all the JSLE patients are atopic but do not have clinical allergy. Therefore, they were not obviously influenced by powerful environmental factors. The high prevalence of atopy may also be associated with immune dysfunction and play an important role in the JSLE disease activity.

In addition, there are evident improvements in SLEDAI-2K score, cumulative glucocorticoid calculated by unit body weight, kinds of immunosuppressor used, complement C3, and NK cell cytotoxicity in JSLE without atopy at the 1st, 3rd, 6th, 12th, 18th, and 24th months of follow-up. The findings showed that patients with JSLE and atopy experienced significantly more severe disease activity and much more time to reach remission. Several studies also indirectly consider this relationship by examining autoimmune disease in relation to allergies and asthma, which showed positive associations [31, 32]. However, some studies report an inverse association of childhood farm exposure to animals and grains with SLE, suggesting the possible operation of hygiene hypothesis [33, 34]. There are some possible explanations for these inconsistent findings. Most of the studies are retrospective and history of transient or mild allergic symptoms is likely to be subject to inaccurate recall. Previous study considered that examining determinants of IgE levels in SLE patients had limited number of patients and controls [35]; thus, their findings are inconclusive. Total IgE levels in allergic individuals may reflect the potential for antigen-specific IgE production associated with hypersensitivity reactions [33,34]. These studies utilize total serum IgE to test IgE-mediated allergies. However, in the present study, we directly use allergen-specific tests or SPT to diagnose atopy. Another limitation of previous studies is the lack of information on current or recent allergic symptoms and allergic phenotype, which may influence disease activity. Allergies may be triggered by exposure to various ubiquitous or episodic exposures to antigens. For the purpose of analyses, the atopy group in the current study was restricted to patients without clinical allergy and antianaphylactic treatment to reduce influences of confounding factors. Most importantly, previous studies did not provide medication data and objective measure (subjective self-report of whether disease was flaring), as well as comprehensive laboratory indicators of inflammation and antibody levels. Most of the studies included adult SLE. No studies have evaluated the influence of co-existing atopy on JSLE disease activity and prognosis.

Both groups experienced an excellent overall response to treatment that may limit the power of the study to detect significant between-group differences. In addition, JSLE patients with atopy had higher disease activity and received more aggressive therapy (more kinds of immunosuppressor used and more dose of glucocorticoid calculated by unit body weight) at the disease onset, which may influence the outcome of JSLE. Thus, we introduced a new measurement “ΔSLEDAI-2K,” which can reflect the change of improvement rate of SLEDAI-2K before and after the follow-up. ΔSLEDAI-2K can also indicate the degree of change of SLEDAI-2K. The ΔSLEDAI-2K was higher in the non-atopic group at the 1st, 3rd, 6th, 12th, 18th, and 24th months (56.95 vs. 43.63%; 75.10 vs. 48.39%; 77.26 vs. 53.59%; 84.10 vs. 69.54%; 82.93 vs. 68.14%; 81.88 vs. 70.00%, respectively; all *p*<0.005; Fig 1). This finding further shows that the JSLE patients with atopy need more time to reach remission and have a poorer outcome. Moreover, fewer patients with atopy reached a decrease of SLEDAI-2K score by 50% at the first month follow-up.

The ultimate treatment decision is made by the physician considering individual characteristics. To avoid the effects of antianaphylactic treatment on disease outcome, we excluded the 8 JSLE patients with clinical allergy. Although all JSLE patients were taking glucocorticoids during the study, the response to medications of the two groups differed significantly. This difference may result from co-existing atopy and the underlying immune dysfunction. Therefore, the results of the current treatment were convincing. Although higher cumulative glucocorticoid calculated by unit body weight and kinds of immunosuppressor were taken in the atopic group during the follow-up, thereby suggesting a difference in the treatment, the ΔSLEDAI-2K rate remained lower during the study in the atopic group. This may imply that co-existent atopy in JSLE patients might complicate the response to therapy. JSLE patients with atopy were treated more aggressively with glucocorticoid and immunosuppressor than the non-atopic group.

To determine outcomes in JSLE patients, we analyzed the number of disease flares and the time to reach the stable condition of the disease at the end of the two-year follow-up. JSLE patients with atopy exhibited significantly higher number of disease flares and longer time to reach disease remission. The knowledge of these associations is important from the therapeutic point of view, as the antihistaminic drugs for atopy may be helpful in the treatment of the disease flares. This study is the first to report this phenomenon and suggests that JSLE patients with atopy may have more severe disease and poorer outcomes compared to JSLE patients without atopy.

However, plausible immunological explanations for the associations between JSLE and co-existing atopy remained to be clarified. In the present study, JSLE patients with atopy at the disease onset had markedly higher total serum IgE and no evident difference in total serum IgE was observed at the end of follow-up. Elevated total serum IgE levels have been described in patients with SLE that are driven by the aberrant production of interferon and self-damaging autoantibodies [35]. Thus, we analyzed the correlations between total serum IgE and SLEDAI-2K at the baseline, 18th month, and end of the follow-up. The total serum IgE levels did not statistically differ with respect to the SLEDAI-2K score (at the baseline, r = 0.16, *p* = 0.147; at the 18th month, r = 0.21, *p* = 0.057; at the end of follow-up, r = -0.03, *p* = 0.81, respectively; Fig 2). Our research was in accordance with recent studies, which suggested that increased serum IgE levels were unrelated to allergic diseases and disease activity in patients with JSLE or adult SLE [34–36]. However, the diagnosis of atopy in our study was based on patients’ specific-IgE or SPT, which is evidently different from the total IgE. SLE patients had increased total IgE serum levels [34]. This increase in total IgE levels was not related to allergic diseases [35], and our results are in line with the hypothesis that high IgE levels can be considered a marker of immune dysregulation [35]. However, several studies suggested that elevated IgE correlates with more severe disease activity and nephritis [36,37]. But these potential associations of SLE with atopic disorders are inconsistent because they propose potential common underlying risk factors or a shared mechanism of allergic and autoimmune phenotype [34,35]. The dysregulation of immune tolerance also results in aberrant Th2 responses and polyclonal activation of B lymphocytes along with the production of autoantibodies, including those of IgE isotype [35,38,39]. Human Th2 responses, characterized by the significance of IL-4, IL-5, IL-10, and IgE, are mainly observed in atopic disease [8]. However, elevated IgE production is also observed in patients with partial T cell immunodeficiencies and autoimmune disease [34, 35, 40]. The most striking observation reported in literature is that elevated IgE production and allergic and autoimmune manifestations frequently occur in patients with partial T cell immunodeficiencies even though elevated IgE levels and autoimmune and inflammatory diseases are commonly associated with hyperactivity of the adaptive immune system [40].

NK cells are a third type of lymphocyte in addition to T and B cells and are an important link between the innate and adaptive immune systems. The major functional properties of NK cells are cytotoxicity and cytokine production [41]. Autoimmune diseases are initiated in steps, as pointed by previous study, including release of self-antigens from the target organ, and finally immune cell homing to the target organ/tissue and subsequent tissue destruction [42]. NK cells can play an important role in all these steps, leading to release of self-antigens, at the level of T-cell priming in secondary lymphoid organs and at the level of the target organ as immunomodulatory or effector cells [41, 43]. Recent findings have suggested that NK cells are few in number and show a dysfunctional phenotype in patients with active SLE [44]. However, no relative data about JSLE and JSLE combined with atopy are available. In the present study, we are the first to determine the decrease of NK cell cytotoxicity and NK cell percentage in JSLE with atopy, which may also be the underlying etiologic causes and pathogenic mechanism of both diseases. This association should be further investigated in a large number of studies.

The pathogenesis of JSLE combined with atopy is speculated to involve a complex interplay between the innate and adaptive immune systems. While key pathogenic features of atopic disorders include an aberrant response to allergens through IgE production by antigen-specific T helper cells and B cells [20, 45]. And both JSLE and atopy are associated with a Th2 cytokine pattern. As T and B helper cells are involved in the pathogenesis of both atopy and autoimmune disease, a positive association is speculated [46]. Many common immune pathways underlying autoimmune disease and atopy, featuring Th2, Th17, Treg, and Toll-like receptors (TLRs) have been the subject of several recent reports [17–20, 47].

Recent evidences have indicated that IL-17 plays a role in the pathogenesis of SLE [48,49]. SLE patients have higher serum levels of IL-17 and IL-23 than healthy controls [50, 51]. Moreover, plasma IL-17 levels are positively correlated with SLE disease activity [48]. Apart from the obvious proinflammatory activities attributed to IL-17, increased production of total IgG, anti-dsDNA IgG, and IL-6 by peripheral blood mononuclear cells of patients with lupus nephritis was observed when cultured with IL-17 [49,52]. These findings suggest that IL-17 may participate in the activation of B cells in patients with SLE [49]. In addition to their role in SLE, Th17 cells also contribute to the development of atopy by promoting IgE class-switch recombination in B cells through stimulation of ε germline transcripts, thereby promoting IgE production [53, 54]. The similarities of the cytokine milieu between SLE and atopy may suggest that SLE and atopic disorders may well co-exist in a given patient and interact with each other. However, it is recognized in a recent study that Th17 cells can acquire a Th1 phenotype (Th17/Th1) [55], evolve into IFN-γ-producing T cells, and secrete the pro-inflammatory cytokines TNF-α, IL-1-β, and IL-17 [53, 55], thereby exacerbating the proinflammatory state of SLE and leading to the persistence of chronic inflammation in the context of autoimmune disease. Taken together, these observations provide immunological support for the viewpoint in some extent that the common disordered immune pathways can permit the co-occurrence of JSLE and atopy and may induce a less favorable outcome for patients with JSLE.

In summary, recent studies are mainly designed to determine the epidemiologic relation of atopy and SLE by questionnaires, consensus, or prevalence surveys and the underlying pathologic intersection of these diseases; their results suggest the rise in the prevalence of both atopy and SLE and a common mechanism leading to a high risk factor for each other [4, 16–20, 34]. Given the fact that previous studies are mainly conducted in adults and quantitative standard and specific description of the disease are lacking, limited data exist regarding the influence of co-existing atopy on JSLE. To date, no unified conclusion has been achieved for the relation between autoimmune and atopic disorders. This research is the first to quantify the disease status by disease indices of wide acceptance and validation, laboratory test results, the statistics of disease flares and relapses, the kinds of immunosuppressor used, and the dose of glucocorticoids, as well as to compare the clinical manifestations, treatment course, and laboratory results of JSLE patients with atopy to those without atopy in a systematic manner.

The present study had limitations. It was a prospective clinical analysis with a relatively small cohort. We also did not detect and quantify relative cytokine activity that may further underline the poorer outcomes of JSLE with concurrent atopy. Trials that incorporate laboratory assessments of these factors are expected to add further weight to the findings discussed here and will be the focus of further study.

In conclusion, our study with 2-year follow-up provides strong prospective clinical data to support the concept of co-existence of JSLE and atopy and emphasizes the potential effect of co-existing atopy on the course and prognosis of JSLE. Our study has observed the adverse impact of co-existing atopy on the prognosis of JSLE and we suggest that co-morbidities should be considered in treating JSLE patients.

## Supporting information

S1 Dataset A standardized data collection protocol was used for all patients. (XLS)

## References

[1] Brunner HI, Huggins J, Klein-Gitelman MS. Pediatric SLE—towards a comprehensive management plan. Nat Rev Rheumatol. 2011; 7(4): 225–233. 10.1038/nrrheum.2011.15 21386795

[2] Ferriani MP, Silva MF, Pereira RM, Terreri MT, Saad Magalhães C, Bonfá E, et al Chronic Spontaneous Urticaria: A Survey of 852 Cases of Childhood-Onset Systemic Lupus Erythematosus. Int Arch Allergy Immunol. 2015; 167(3): 186–192. 10.1159/000438723 26329010

[3] Borchers AT, Naguwa SM, Shoenfeld Y, Gershwin ME. The geoepidemiology of systemic lupus erythematosus. Autoimmu Rev. 2010; 9(5): A277–A287. 10.1016/j.autrev.2009.12.008 20036343

[4] Huang J, Yao T, See L. Prevalence of pediatric systemic lupus erythematosus and juvenile chronic arthritis in a Chinese population: a nation-wide prospective population-based study in Taiwan. Clin Exp Rheumatol. 2004; 22(6): 776–780. 15638056

[5] Kurahara DK, Grandinetti A, Fujii LL, Tokuda AA, Galario JA, Han MJ, et al Visiting consultant clinics to study prevalence rates of juvenile rheumatoid arthritis and childhood systemic lupus erythematosus across dispersed geographic areas. J Rheumatol. 2007; 34(2): 425–429. 17295431

[6] Yap DYH, Lai KN. Cytokines and their roles in the pathogenesis of systemic lupus erythematosus: from basics to recent advances. J BioMed Biotechnol. 2010; 2010:36508. 10.1155/2010/365083 PMC2866250 20467470

[7] Thomsen SF. Epidemiology and natural history of atopic diseases. Eur Clin Respir J. 2015 3 24; 2. eCollection 2015. 10.3402/ecrj.v2.24642 PMC4629767 26557262

[8] Upham JW, Holt PG. Environment and development of atopy. Cur Opin Allergy Clin Immunol. 2005; 5(2): 167–172. 10.1097/01.all.0000162310.79555.ed 15764908

[9] Asher MI, Montefort S, Björkstén B, Lai CK, Strachan DP, Weiland SK, et al Worldwide time trends in the prevalence of symptoms of asthma, allergic rhinoconjunctivitis, and eczema in childhood: ISAAC Phases One and Three repeat multicountry cross-sectional surveys. Lancet 2006; 368(9537): 733–743. 10.1016/S0140-6736(06)69283-0 16935684

[10] Schuh C, Fritscher LG, Chapman KR, Fritscher CC. The prevalence of asthma and atopy in schoolchildren from Porto Alegre, Brazil, has plateaued. Respir Med 2015; 109(3): 308–311. 10.1016/j.rmed.2015.01.014 25683031

[11] Heinrich J, Popescu MA, Wjst M, Goldstein IF, Wichmann HE. Atopy in children and parental social class. Am J Public Health. 1998; 88(9): 1319–1324. 9736870 10.2105/ajph.88.9.1319 PMC1509081

[12] Leung R, Ho P, Lam CK, Lai CW. Sensitization to inhaled allergens as a risk factor for asthma and allergic diseases in Chinese population. J Allergy Clin Immunol. 1997; 99(5): 594–599. 9155823 10.1016/s0091-6749(97)70018-6

[13] Hemminki K, Li X, Sundquist J, Sundquist K. Subsequent autoimmune or related disease in asthma patients: clustering of diseases or medical care? Ann Epidemiol. 2010; 20(3): 217–222. 10.1016/j.annepidem.2009.11.007 20036578

[14] Ehlers S, Kaufmann SH. Infection, inflammation, and chronic diseases: consequences of a modern lifestyle. Trends Immunol. 2010; 31(5): 184–190. 10.1016/j.it.2010.02.003 20399709

[15] Bach J-F. The effect of infections on susceptibility to autoimmune and allergic diseases. N Engl J Med. 2002; 347(12): 911–920. 10.1056/NEJMra020100 12239261

[16] Hsiao YP, Tsai JD, Muo CH, Tsai CH, Sung FC, Liao YT, et al Atopic diseases and systemic lupus erythematosus: an epidemiological study of the risks and correlations. Int J Environ Res Public Health. 2014; 11(8): 8112–8122. 10.3390/ijerph110808112 25111878 PMC4143852

[17] Wu LC, Hwang CY, Chung PI, Hua TC, Chen YD, Chu SY, et al Autoimmune disease comorbidities in patients with atopic dermatitis: a nationwide case–control study in Taiwan. Pediatr Allergy Immunol. 2014; 25(6): 586–592. 10.1111/pai.12274 25223227

[18] Maas T, Nieuwhof C, Passos VL, Robertson C, Boonen A, Landewé RB, et al Transgenerational occurrence of allergic disease and autoimmunity: general practice-based epidemiological research. Prim Care Respir J. 2014; 23(1): 14–21. 10.4104/pcrj.2013.00108 24449016 PMC6442276

[19] Guo R, Cao L, Kong X, et al Atopy in children with the enthesitis-related arthritis (ERA) subtype of juvenile idiopathic arthritis is associated with a worse outcome. Eur J Pediatr. 2015; 174(11): 1441–1450. 10.1007/s00431-015-2553-1 25935591

[20] Shah A. The pathologic and clinical intersection of atopic and autoimmune disease. Curr Allergy Asthma Rep. 2012; 12(6): 520–529. 10.1007/s11882-012-0293-0 22898881

[21] Hochberg MC. Updating the American College of Rheumatology revised criteria for the classification of systemic lupus erythematosus. Arthritis Rheum. 1997; 40(9): 1725. 10.1002/art.1780400928 9324032

[22] Rao V, Gordon C. Advances in the assessment of lupus disease activity and damage. Curr Opin Rheumatol. 2014; 26(5): 510–519. 10.1097/BOR.0000000000000085 25010438

[23] Custovic A, Lazic N, Simpson A. Pediatric asthma and development of atopy. Curr Opin Allergy Clin Immunol. 2013; 13(2): 173–180. 10.1097/ACI.0b013e32835e82b6 23385287

[24] Korzeniewski C, Callewaert DM. An enzyme-release assay for natural cytotoxicity. J Immunol Methods. 1983; 64(3): 313–320. 6199426 10.1016/0022-1759(83)90438-6

[25] Habibi S, Saleem M, Ramanan A. Juvenile systemic lupus erythematosus: review of clinical features and management. Indian pediatr. 2011; 48(11): 879–887. 22711146 10.1007/s13312-011-0143-5

[26] Kitaba S, Matsui S, Iimuro E, Nishioka M, Kijima A, Umegaki N, et al Four cases of atopic dermatitis complicated by Sjogren's syndrome: link between dry skin and autoimmune anhidrosis. Allergol Int. 2011; 60(3): 387–391. 10.2332/allergolint.10-CR-0265 21364310

[27] Wei L, Chen H, Yu H, Zhijun Z, Sundell Jan. Associations between indoor environmental smoke and respiratory symptoms among preschool children in Shanghai, China. Chin Sci Bull. 2013; 58(34):4211–4216.

[28] Chen H, Yu H, Wei L, Zhijun Z, Sundell Jan. Pet-keeping and its impact on asthma and allergies among preschool children in Shanghai, China. Chin Sci Bull. 2013; 58(34):4203–4210.

[29] Confino-Cohen R, Chodick G, Shalev V, Leshno M, Kimhi O, Goldberg A. Chronic urticaria and autoimmunity: associations found in a large population study. J Allergy Clin Immunol. 2012; 129(5): 1307–1313. 10.1016/j.jaci.2012.01.043 22336078

[30] Rudwaleit M, Andermann B, Alten R, Sörensen H, Listing J, Zink A, et al Atopic disorders in ankylosing spondylitis and rheumatoid arthritis. Ann Rheum Dis. 2002; 61(11): 968–974. 10.1136/ard.61.11.968 12379517 PMC1753933

[31] Sheikh A, Smeeth L, Hubbard R. There is no evidence of an inverse relationship between TH2-mediated atopy and TH1-mediated autoimmune disorders: Lack of support for the hygiene hypothesis. J Allergy Clin Immunol. 2003; 111(1): 131–135. 12532108 10.1067/mai.2003.8

[32] Simpson CR, Anderson WJ, Helms PJ, Taylor MW, Watson L, Prescott GJ, et al Coincidence of immune-mediated diseases driven by Th1 and Th2 subsets suggests a common aetiology. A population-based study using computerized general practice data. Clin Exp Allergy. 2002;32(1):37–42. 12002734 10.1046/j.0022-0477.2001.01250.x

[33] Parks CG, Cooper GS, Dooley MA, Park MM, Treadwell EL, Gilkeson GS. Childhood agricultural and adult occupational exposures to organic dusts in a population-based case-control study of systemic lupus erythematosus. Lupus. 2008;17(8):711–719. 10.1177/0961203308089436 18625648

[34] Parks CG, Biagini RE, Cooper GS, Gilkeson GS, Dooley MA. Total serum IgE levels in systemic lupus erythematosus and associations with childhood onset allergies. Lupus. 2010;19(14):1614–1622. 10.1177/0961203310379870 20937624

[35] Liphaus BL, Jesus AA, Silva CA, Coutinho A, Carneiro-Sampaio M. Increased IgE serum levels are unrelated to allergic and parasitic diseases in patients with juvenile systemic lupus erythematosus. Clinics (Sao Paulo). 2012;67(11):1275–1280. 23184203 10.6061/clinics/2012(11)09 PMC3488985

[36] Elkayam O, Tamir R, Pick AI, Wysenbeek A. Serum IgE concentrations, disease activity, and atopic disorders in systemic lupus erythematosus. Allergy. 1995;50(1):94–96. 7741196

[37] Rebhun J, Quismorio F Jr, Dubois E, Heiner DC. Systemic lupus erythematosus activity and IgE. Ann Allergy. 1983;50(1):34–36. 6849518

[38] Atta AM, Sousa CP, Carvalho EM, Sousa-Atta ML. Immunoglobulin E and systemic lupus erythematosus. Braz J Med Biol Res. 2004;37(10):1497–1501. 15448870 10.1590/s0100-879x2004001000008

[39] Atta AM, Santiago MB, Guerra FG, Pereira MM, Sousa Atta ML. Autoimmune response of IgE antibodies to cellular self-antigens in systemic Lupus Erythematosus. Int Arch Allergy Immunol. 2010;152(4):401–406. 10.1159/000288293 20197682

[40] Liston A, Enders A, Siggs OM. Unravelling the association of partial T-cell immunodeficiency and immune dysregulation. Nat Rev Immunol. 2008;8(7):545–558. 10.1038/nri2336 18551129

[41] Flodström-Tullberg M, Bryceson YT, Shi FD, Höglund P, Ljunggren HG. Natural killer cells in human autoimmunity. Curr Opin Immunol. 2009;21(6):634–640. 10.1016/j.coi.2009.09.012 19892538

[42] Ji H, Korganow AS, Mangialaio S, Höglund P, André I, Lühder F, et al Different modes of pathogenesis in T-cell-dependent autoimmunity: clues from two TCR transgenic systems. Immunol Rev. 1999;169:139–146. 10450514 10.1111/j.1600-065x.1999.tb01312.x

[43] Shi FD, Van Kaer L. Reciprocal regulation between natural killer cells and autoreactive T cells. Nat Rev Immunol. 2006;6(10):751–760. 10.1038/nri1935 16998508

[44] Spada R, Rojas JM, Barber DF. Recent findings on the role of natural killer cells in the pathogenesis of systemic lupus erythematosus. J Leukoc Biol. 2015;98(4):479–487. 10.1189/jlb.4RU0315-081RR 26216938

[45] Tjota MY, Hrusch CL, Blaine KM, et al Signaling through FcRγ-associated receptors on dendritic cells drives IL-33–dependent T H 2-type responses. J Allergy Clin Immunol. 2014; 134(3): 706–713.e8. 10.1016/j.jaci.2014.06.013 25088053 PMC4149927

[46] Rabin R, Levinson A. The nexus between atopic disease and autoimmunity: a review of the epidemiological and mechanistic literature. Clin Exp Immunol. 2008; 153(1): 19–30. 10.1111/j.1365-2249.2008.03679.x 18505431 PMC2432093

[47] Cosmi L, Maggi L, Santarlasci V, Liotta F, Annunziato F. T helper cells plasticity in inflammation. Cytometry A. 2014; 85(1): 36–42. 10.1002/cyto.a.22348 24009159

[48] Garrett-Sinha LA, John S, Gaffen SL. IL-17 and the Th17 lineage in systemic lupus erythematosus. Curr Opin Rheumatol. 2008;20(5):519–525. 10.1097/BOR.0b013e328304b6b5 18698171

[49] Wong CK, Ho CY, Li EK, Lam CW. Elevation of proinflammatory cytokine (IL-18, IL-17, IL-12) and Th2 cytokine (IL-4) concentrations in patients with systemic lupus erythematosus. Lupus. 2000;9(8):589–593. 10.1191/096120300678828703 11035433

[50] Wong CK, Lit LC, Tam LS, Li EK, Wong PT, Lam CW. Hyperproduction of IL-23 and IL-17 in patients with systemic lupus erythematosus: implications for Th17-mediated inflammation in auto-immunity. Clin Immunol. 2008;127(3):385–393. 10.1016/j.clim.2008.01.019 18373953

[51] Nalbandian A, Crispín JC, Tsokos GC. Interleukin-17 and systemic lupus erythematosus: current concepts. Clin Exp Immunol. 2009;157(2):209–15. 10.1111/j.1365-2249.2009.03944.x 19604260 PMC2730846

[52] Dong G, Ye R, Shi W, Liu S, Wang T, Yang X, et al IL-17 induces autoantibody overproductionand peripheral blood mononuclear cell overexpressionof IL-6 in lupus nephritis patients. Chin Med J (Engl). 2003;116(4):543–548. 12875719

[53] Quan SH, Zhang YL, Han DH, Iwakura Y, Rhee CS. Contribution of interleukin 17A to the development and regulation of allergic inflammation in a murine allergic rhinitis model. Ann Allergy Asthma Immunol. 2012;108(5):342–350. 10.1016/j.anai.2012.02.014 22541406

[54] Milovanovic M, Drozdenko G, Weise C, Babina M, Worm M. Interleukin-17A promotes IgE production in human B cells. J Invest Dermatol. 2010;130(11):2621–2628. 10.1038/jid.2010.175 20596087

[55] Nistala K, Adams S, Cambrook H, Ursu S, Olivito B, de Jager W, et al Th17 plasticity in human autoimmune arthritis is driven by the inflammatory environment. Proc Natl Acad Sci U S A. 2010;107(33):14751–14756. 10.1073/pnas.1003852107 20679229 PMC2930428

